# Fast and Reliable Alternative to Encoder-Based Measurements of Multiple 2-DOF Rotary-Linear Transformable Objects Using a Network of Image Sensors with Application to Table Football

**DOI:** 10.3390/s20123552

**Published:** 2020-06-23

**Authors:** Matevž Bošnak, Gregor Klančar

**Affiliations:** Faculty of Electrical Engineering, University of Ljubljana, 1000 Ljubljana, Slovenia; gregor.klancar@fe.uni-lj.si

**Keywords:** translation-rotation measurement, visual encoder, data fusion

## Abstract

Simultaneous determination of linear and angular positions of rotating objects is a challenging task for traditional sensor applications and a very limited set of solutions is available. The paper presents a novel approach of replacing a set of traditional linear and rotational sensors by a small set of image sensors. While the camera’s angle of view can be a limiting factor in the tracking of multiple objects, the presented approach allows for a network of image sensors to extend the covered area. Furthermore, rich image data allows for the application of different data processing algorithms to effectively and accurately determine the object’s position. The proposed solution thus provides a set of smart visual encoders emulated by an image sensor or a network of image sensors for more demanding spatially distributed tasks. As a proof of concept, we present the results of the experiment in the target application, where a 1.6 MP image sensor was used to obtain sub-degree angular resolution at 600 rpm and thus exceeding the design parameters and requirements. The solution allows for a compact, cost-effective, and robust integration into the final product.

## 1. Introduction

**Figure 1:**
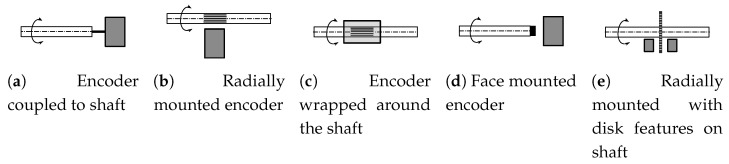
Common encoder mounting types.

Linear and rotary position sensors are an essential part of different actuation systems and there are not only numerous variations of the proposed solutions, but also several real-world implementations. These rely on different physics principles, varying from being mechanical, electro-magnetic (e.g., resistive, capacitive or magnetic) to optical. In most cases, linear and rotary position sensors can not be combined directly to measure the linear and rotary position of an object—while shaft rotation sensors are regarded as COTS (Common Off-The-Shelf) components, most types require the shaft to have no or very limited linear play [1,2]. The limitation comes from the fact that the sensor consists of two parts, one coupled to the rotating body and the other fixed to the housing. Compliance of the rotating body in both the axial and radial axis can compromise the ability of the system to provide accurate feedback [3]. Although most types rely on a disk-like feature installed on the rotating body, certain optical, magnetic and capacitive sensor types allow the rotating features to be extended in the axial direction over the length of the body and can thus tolerate linear play of the shaft (Figure 1). Similarly, the linear position sensors operate by measuring the distance between two sensor features and most of them can tolerate the rotational motion of the otherwise linearly displaced object. Most common angular encoder types with corresponding mounting options have been summarized in Table 1. Unfortunately, there is a very limited subset of available solutions that would be compatible with rotational and linear motion and even more limited subset of solutions that support measurement of position of both, the area we are proposing the solution for in this paper.

There are two main categories for the position sensors—relative and absolute. Relative sensors provide information on positional displacement between two consecutive instances and the integration step of these measurements is needed to produce the position itself. The result is ambiguous due to the unknown starting position. This is partly solved with the use of absolute encoders that provide information on the absolute position of the tracked object. Although some applications couple the sensor itself with the processing logic and battery backup power to allow the relative encoders to behave as absolute ones, we will focus on the sensor types that itself can determine the absolute position. In the case of rotational absolute encoders, it is a common practice that term absolute position relates to one rotation only, that is, an angle in the range of $\left[0,2\pi \right)$. In some cases, it is beneficial to use other ranges, that is, for electronically commutated motors [4].

The most common implementations of the angular absolute encoders code the angular position with binary values, defined by different sequences of features on the rotating component. The resolution of such sensors is usually limited by the spacing of the features. On the other hand, interpolation-based methods are not limited by the resolution of the features, but we find the application limited to incremental sin/cos encoders, resolvers [5] and other niche applications [6]. The prevailing data encoding approach of binary-features based solutions is in the use of single-distance codes, in reference to the Hamming distance of 1 between adjacent codes. This results in well defined angular positions that are mostly immune to switching delays of the sensing parts (historically, the encoders were mechanical devices, where individual signal contacts were subjected to bouncing and other switching anomalies). Gray code is a familiar term in absolute encoders and serves as a basis for a large set of encoder implementations. Single track Gray code absolute encoders [7] allow for multiple sensing elements to replace multiple tracks of the encoded data with a specially designed single track data. Although it is not possible to distinguish $2^{n}$ positions with *n* sensors on a single track, it is possible to distinguish close to that many. This approach is similar to the one used in pseudorandom position encoder [8], where the pseudorandom sequence uniquely defines each step of the position data. Another approach uses multiple tracks and multiple sensors, presented in Reference [9], where the resolution of the position is still defined by the granularity of the coded pattern.

Increasing the number of (optical) sensing elements in such applications, naturally leads to camera-based solutions. In Reference [10], the authors present the approach that closely resembles idea from Reference [9], but with the use of a CCD sensor. As a slight modification, the authors of Reference [11] present the absolute rotary encoder that uses a CMOS sensor and barcode-like features radially arranged on a disk. Another subset of solutions employs fast feature tracking capability of optical mouse sensor [12], while the authors of Reference [13] present an approach of using the image acquisition capabilities of the optical mouse sensor to build an absolute rotary encoder. However, the nature of camera-based solutions allows for more innovative approaches, freedom in feature selection, and thus more flexible applications. Camera is often used in sensor fusion estimators to improve localization results as in References [14,15]. In Reference [16] the term of a visual encoder is presented, where authors describe the idea of robustly and precisely measuring the rotation angle of an object by tracking an RGB color pattern aligned on the rotor and tracked via high-speed RGB vision system. Similarly, the authors of Reference [17] employ different color gradients to determine the rotational angle. The authors of Reference [18] present the solution for data processing to improve on capture image contrast and thus improve both the low light and the high-speed performance.

Reference [19] presents the application of the aforementioned approaches using color gradients and photoelectric sensors and introduces the capability of tracking the linear and angular rotor position simultaneously. A specially designed color pattern allows for the distinction between axial and radial patterns by color masking. Object tracking using the camera capture system traditionally allows for a 2-D position and angle, which is usually limited to the angular axis that coincides with the plane normal vector, as in Reference [20].

The solution in this paper combines these ideas into a novel solution for simultaneous tracking of the object’s linear and angular position using a high-speed vision system. The system is capable of contactless tracking of multiple objects and thus presents a cost-effective and very compact solution. In this work we present the overall system design, components selection and placement, image processing steps, and the target application. The performance of the system is evaluated and presented in the final part of the paper.

## 2. Operating Principle

The underlying concept is in replacing physical sensors with a set of image-based ones, smart sensors rendered by the image processing, and data fusion algorithms. This approach allows us to combine the traditionally separated measurements of translation and rotation into a single smart sensor unit. The suggested approach addresses tracking of an elongated cylindrical object’s (referred to as a rod) bounded linear motion along the main principal axis of rotation (referred to as translation) and unbounded rotation motion around the same axis (referred to as rotation). However, the approach can also be generalized to any object that contains the noted cylindrical part and does not occlude it in terms of the camera’s field of view. The object is outfitted with a marker, an artificially created pattern that is wrapped around the object, which allows the image recognition system to locate its position and orientation in the image, described in Section 2.2.

The translation of the target with the marker will result in a change of its apparent position in the camera image, while the rotation of the target will only change the appearance of it. Moreover, the translation is bounded to one axis and all possible apparent positions form a line along that axis. The global camera image can thus be segmented into multiple areas of interest, each corresponding to a tracked object. A particular area is then first analyzed to detect the position of the marker and thus define the object’s translation. Second processing step positions the rotation decoder over the target and the rotation is first estimated using the Gray code pattern, followed by the fine angular position determination using the phase-detection over the least significant bit area of the Gray code.

The presented idea is based on using a network of synchronized color video cameras, overlooking the tracked objects, as will be presented in Section 3. In this paper we will focus on an application, where the tracked object does not leave the field of view of a single camera. Multiple objects in the global camera image can be tracked at the same time using the approach. Moreover, a network of image sensors covers the larger area, containing a set of even more objects, allowing sensor fusion algorithms to be employed to improve the accuracy of the results for the objects from the overlapping set.

The design requirements for the proposed system were governed by the target application, which is presented in the final part of the paper. The required measurement accuracy was approximately 1 mm for the translation and 3 degrees for the rotation, while the capture frequency of at least 100 Hz was determined to be necessary for the successful implementation of the control system in the target application. It was seen beneficial if measurement resolution is better than the specified accuracy figures. An important aspect of the usability of the solution is also its robustness to illumination variations—loosing the tracked target data due to non-uniform lighting conditions is detrimental to the application and thus unwanted. The proposed system uses a compact LED-based linear fixture and can operate with or without additional lights in the environment.

To summarize, our approach requires a camera with image-capture frequency chosen based on application specification. Its location needs to provide unobstructed view of the tracked object, while its resolution is chosen to guarantee reliable recognition of the marker pattern (as stated in Section 2.2). Specifications for the camera system used in this work are detailed in Section 3.

### 2.1. Camera Setup, Image Capture, and Processing

**Figure 2:**
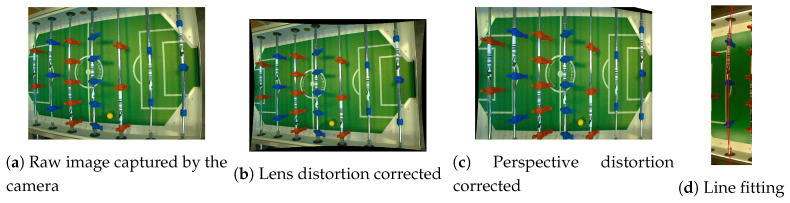
Image processing chain.

Each camera is processed individually in its own processing pipeline and the separate results are joined in the common position filtering step. Processing in each pipeline starts with the image being captured and converted from Bayer to RGB color space (a sample captured image in RGB color space is shown below in Figure 2a). Synchronization of the image capture step among multiple cameras in the network is accomplished via a hardware clock signal that is generated by one of the cameras.

Image-based object tracking is very active research field and different approaches to the solution have been proposed. Most of these solutions propose a two-step approach, suggesting a more complex and slower object detection for initialization of the object tracking algorithm. This results in improved performance over constantly running object detection, but requires a reliable failure detection and recovery [21,22]. The reliability of the detector and tracker is of paramount importance for automotive applications [23], where incorrect object position or the orientation can result in dangerous reaction of the automated driving system. Other proposed solutions use object model for robust tracking in complex environments [24], the idea that is used and enhanced in our approach. The highly predictable environment grants the use of application-specific object model, that combines the object with the camera distortions.

Traditionally, camera lens distortion correction (Figure 2b) and perspective transform (Figure 2c) would be applied to the image, but these two operations need to be applied to the whole image and have a heavy computational footprint. In order to achieve target high update frequency (e.g., 100 Hz or more) of the entire system, the approach must be optimized since the regular implementation of these transformation algorithms in the OpenCV library takes roughly 20 ms to process a single image on a desktop PC.

**Figure 3:**
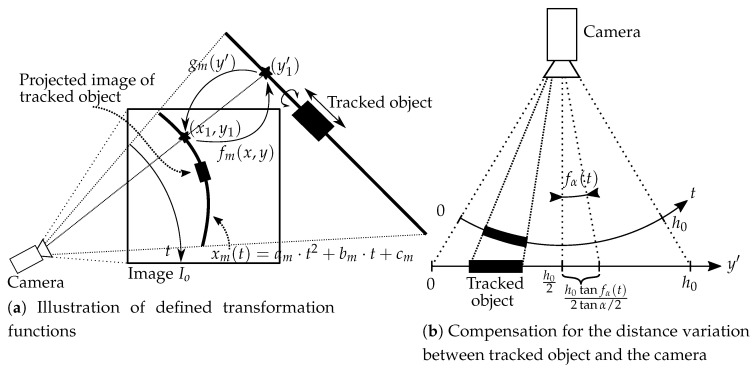
Ilustration of the described approach of converting data between pixel space of image $I_{o}$ and line space of $L_{m}$.

Instead, we identify pixels of interest on the original image and extract only those for further processing. Let us define the transform function $f_{m}\left(x,y\right)$ that will extract pixels from the original two-dimensional color image $I_{o}$ into the one-dimensional set of color pixels (a line) $L_{m}$ (each pixel is represented with a 24-bit color value) for line *m*, written as $f_{m}\left(x,y\right):I_{o}\to L_{m}$. Let us first define the parameter $y^{\prime }$ as the position on the line $L_{m}$ and the inverse function of $g_{m}\left(y^{\prime }\right)$ that provides a look-up relation for each pixel of the one-dimensional line pixel set in the original image (as illustrated in Figure 3a). The inverse function $g_{m}\left(y^{\prime }\right)$ describes the expected trajectory of the target in the image during the translation. Let the working parameter $t\in \left[0,h_{0}\right]\cap Z$ be the height coordinate in the image ($h_{0}=1080$ pixels for camera used in our setup). We can then find a set of $y^{\prime }$, *x* and *y* for each value of the parameter *t* between 0 and the image vertical dimension $h_{0}$. In order to emphasize speed over accuracy at this step, no interpolation method will be used in the $f_{m}\left(x,y\right)$ or its reverse definition.

Since the target trajectory gets distorted by the effect of the camera lens, the mangled trajectory will be estimated with a cubic function in the distorted image. We can define the function $x_{m}\left(t\right)=a_{m}\cdot t^{2}+b_{m}\cdot t+c_{m}$, where parameters $a_{m}$, $b_{m}$ and $c_{m}$ are selected during the camera calibration process by fitting a cubic curve $x_{m}\left(t\right)$ to the distorted appearance of a straight target object in the original image (Figure 2d). Since we assume that there is no rotation around camera viewing axis, that is, the camera’s *x*-axis is always perpendicular to the reference (horizontal) surface normal vector, we will define an additional function $f_{\alpha }\left(t\right)$ as

(1) $$f_{\alpha }\left(t\right)=\frac{t-h_{o}/2}{h_{o}}\cdot \alpha ,$$

where α defines the camera’s view angle (53.2deg for the camera used in the setup) and $h_{o}=1080$ (the image height in pixels). The function $f_{\alpha }\left(t\right)$ compensates for the projection error (as shown in Figure 3b). The reverse function $g_{m}$ is then defined as a map

(2) $$g_{m}\left(\left\lfloor \frac{h_{0}}{2}\left(1+\frac{\tan f_{\alpha }\left(t\right)}{\tan\alpha /2}\right)\right\rfloor \right)\leftarrow I_{o}\left[x_{m}\left(t\right),t\right].$$

The map from Equation (2) can be calculated in advance for each target object m=1…8 and then used as a very fast look-up table operation.

### 2.2. Selecting Marker Pattern

**Figure 4:**
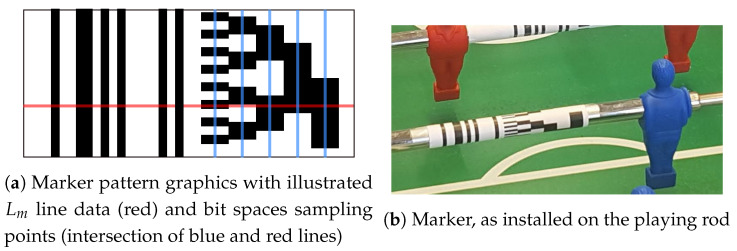
Marker pattern.

A unique marker pattern was selected to achieve two main functions of the system—determination of linear and angular positions. It is one of the most important components of the proposed system since it enables efficient and accurate detection by the computer vision system in order to determine the 2-D positional data. The marker (Figure 4a) is wrapped around the target as shown in Figure 4b and comprises two distinct parts—a 1-D barcode (left 6 stripes) and a pattern based on Gray-code (right, branch-like structure). The two parts can be positioned next to each other or separated by a fixed distance (not affected by the translation of the target).

**Figure 5:**
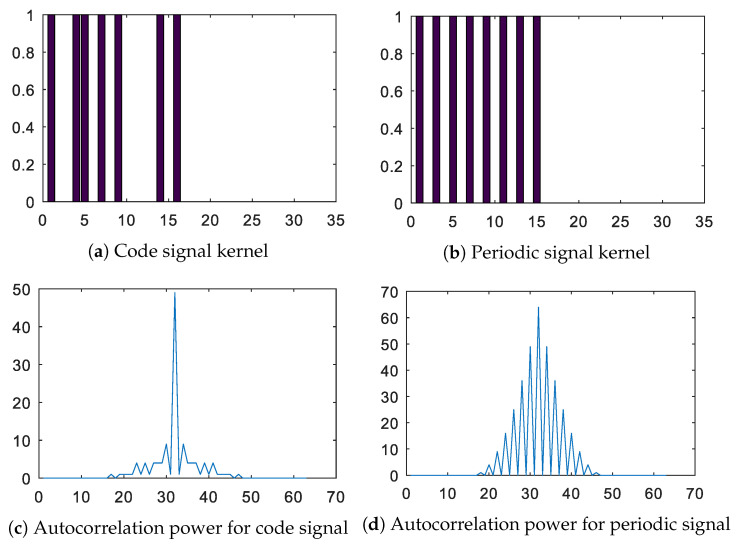
Code signal kernel and its autocorrelation power in comparison to a periodic/repetitive signal. Selected unique code can be robustly detected using correlation because of distinctive peak obtained in its autocorrelation result.

The stripped barcode section was selected to comply with multiple criteria, mostly dealing with the complexity and reliability of the detection algorithm in various camera angles and lighting conditions. Relatively large dimensions of the stripes support the operation under various camera angles and distances, while high-contrast enhances reliability under various lighting conditions. The important part of the barcode is in a non-repeating sequence of bars and spaces, which can be represented by a 16-bit code kernel M(i) with a binary value of 1001101010000101 (illustrated in Figure 5a). In comparison with a periodic sequence of stripes and spaces, the position of the coded sequence in the line data $L_{m}$ can be decisively detected, which is due to the more distinctive peak in the data correlation result [25] (as shown with the auto-correlation power of the coded and periodic barcode signals). Although different code sequences can be used with the same effect, the code pattern is fixed in the presented application for all targets. This is due to the fact that the target trajectories in regards to the camera are known in advance and there is no ambiguity in target identification that would need to be addressed.

The second part of the marker is based on the Gray-code pattern and is intended for determining the rotation angle of the target. A Gray code is a code assigned to each of a contiguous set of angular positions a combination of symbols (coded value) such that no two coded values are identical and each two adjacent coded values differ by exactly one symbol (bit).

The pattern consists of 5 bit spaces, each of them defined by a specific frequency of black and white stripes—bit space 0 contains 8 black and 8 white bars, with each next bit space containing half of the stripes and shifted by 90 degrees in pattern phase (Figure 4a). Bit spaces 3 and 4 have both one pair of black and white stripes. When the pattern is sampled in each bit space along the line data $L_{m}$, a digital, 5-bit angular code is generated. There are 32 distinct values for the obtained result, which corresponds to 360/32≈11 degrees. That does not meet the specified resolution in the project requirements yet, however, this will be later addressed using the phase-detection step (explained in Section 2.5) with sub-degree resolution.

### 2.3. Correlation Step

**Figure 6:**
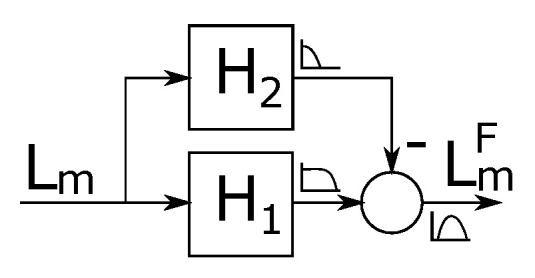
Line data filtering with two low-pass filters.

In order to successfully apply the correlation function in various lighting conditions, the extracted line data $L_{m}\left(j\right)$ must first be filtered with a high-pass filter. High-pass filter removes the lightness gradients across the data due to uneven lighting, which is impossible to control outside the synthetic environment. Additionally, a low-pass filter is applied to the image to reduce the pixel noise. Since high-pass filter can be constructed using the low-pass filter with the use of the analogy, we have implemented the filtering system with two low-pass filters as shown in Figure 6.

**Figure 7:**
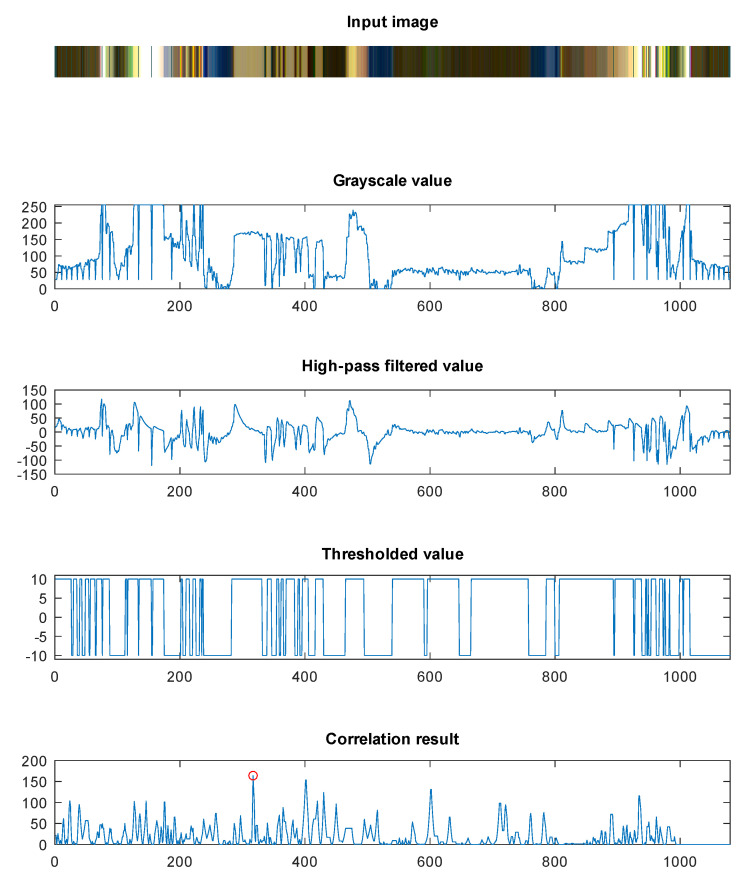
Results of line data analysis and correlation.

Filters $H_{1}$ and $H_{2}$ are discrete IIR (Ininite Impulse Response) first-order low-pass filters with the following equation

(3) $$H\left(z\right)=\frac{\left(1-f\right)z^{-1}}{1-fz^{-1}},$$

where f=−T/(T+1) and the value of *T* is selected for each of the filters separately, as $T_{H_{1}}=0.5$ for high roll-off frequency and $T_{H_{2}}=10$ for low roll-off frequency. The resulting signal $L_{m}^{F}$ is then binarized using hysteresis thresholding operation (results are shown in third and the forth line of Figure 7). This operation processes element by element from the filtered line data $L_{m}^{F}$ to produce thresholded line data $L_{m}^{T}$ with the following rule

(4) $$L_{m}^{T}\left(i\right)=\left\{\begin{array}{cc} 0, & \mathrm{if}\;L_{m}^{F}\left(i\right)<P_{low}\;\mathrm{or}\;\left[L_{m}^{T}\left(i-1\right)=0\;\mathrm{and}\;L_{m}^{F}\left(i\right)<P_{high}\right]\\ 1, & \mathrm{if}\;L_{m}^{F}\left(i\right)>P_{high}\;\mathrm{or}\;\left[L_{m}^{T}\left(i-1\right)=1\;\mathrm{and}\;L_{m}^{F}\left(i\right)>P_{low}\right] \end{array}\right.,$$

where thresholds $P_{high}=-P_{low}=8$ are affected mostly by the amount of noise in the filtered line signal and were selected based on manual optimization. The result of this operation is cleaner binary signal generated from the high-pass filtered pixel data.

In the next step the linear position of the marker sequence M(i) is found in the line data $L_{m}^{T}\left(j\right)$ for the target *m*. This is accomplished by evaluating the cross-correlation function between the signals

(5) $$C\left(k\right)=\left(M★L_{m}^{T}\right)\left(k\right)=\sum_{i}\frac{1}{\sigma_{M}\sigma_{L}}\left(M\left(i\right)-\mu_{M}\right)\left(L_{m}^{T}\left(i+k\right)-\mu_{L}\right),$$

where $\sigma_{M}$, $\sigma_{L}$ are the standard deviations of signals *M* and $L_{m}^{T}$ and $\mu_{M}$, $\mu_{L}$ are averages of *M* and *L*, respectively. We are interested in the position of the peak in the correlation result, the value of $p_{m}={arg max}_{k}{\left(C(k)\right)}^{2}$, which defines the position of the marker sequence in the image (as illustrated by the fifth line in Figure 7).

### 2.4. Angular Position

The sampling of the marker pattern, that is containing the Gray-code encoded angular position, is defined by a set of parameters $O_{m}$ (offset distance in pixels between the origin of the marker sequence M(i) and the origin of the angular code pattern), $N_{b}=5$ (number of decoded bits) and $S_{m}$ (spacing between bit spaces in pixels). Parameters $O_{m}$ and $S_{m}$ are camera-position dependent and are determined for each target individually during camera calibration procedure. Once the linear position of the target $p_{m}$ is determined, a subset of line data $B_{m}\subset L_{m}$ is extracted from $L_{m}\left(j\right)$ for $j=p_{m}+O_{m},\ldots ,p_{m}+O_{m}+N_{b}\cdot S_{m}$.

Since the Gray code decoder expects a binary sequence, the pattern data must be sampled and binarized. Sampled data is first analyzed to determine the lower and upper values of grayscale intensity for sampled data in $B_{m}$, $T_{min}=\min\left\{30,B_{m}\right\}$ and $T_{max}=\max\left\{100,B_{m}\right\}$. An adaptive binarization is then employed using the threshold set to $(T_{min}+T_{max})/2$ and result sampled from $B_{m}$ in the center of each bit space at $i=3S_{m}/2,5S_{m}/2,\ldots ,\left(N_{b}+0.5\right)S_{m}$ and the sample’s grayscale value (B(i)) is binarized to obtain the binary code value $C_{m}$. The absolute angular position $\alpha_{m}$ is then obtained using the look-up table for the Gray code decoder (decoding table is provided in Table 2).

As noted, the resolution of the results obtained using this method (11.25 degrees) does not yet meet the initial project requirements and additional refinement of the results is necessary by the use of phase detection, explained in Section 2.5.

### 2.5. Angular Position Interpolation

The proposed approach combines the idea of interpolation used in the sin/cos resolvers [26] and Gray code absolute encoders with the aim to increase the encoder resolution and improve its performance in the presence of the noise in the captured image. We analyze the area of the first bit of the Gray code and convert the pixel series domain into a frequency domain. Then, we observe the phase at the expected frequency of the data (defined by a distance between black and white stripes in the image).

**Figure 8:**
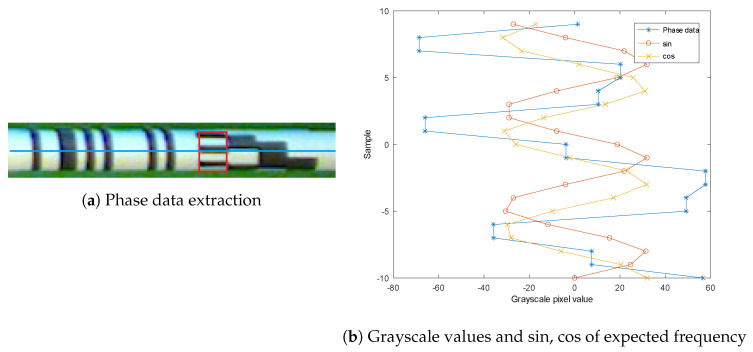
Phase data analysis.

First, additional image data $D_{m}\left(i\right)$ needs to be extracted from $I_{o}$—if the $L_{m}$ data is primarily extracted in the horizontal direction in the image data, the phase data is extracted perpendicular to that (vertical axis), as shown in Figure 8a and marked with a red rectangle. Since the diameter of the target in the captured image is approx. 25 pixels, we take $N_{p}=10$ pixels in each direction from rod-central line (Figure 8a). Grayscale values of the extracted pixels are shown in Figure 8b. Because only the signal phase $\phi_{m}$ must be determined at one specific frequency (defined by signal period $\hat{T}$), the discrete Fourier transform can be simplified into expression

(6) $$\phi_{m}=arctan2\left(\sum_{i=-N_{p}}^{N_{p}}D_{m}\left(i\right)\cdot \sin2\pi i/\hat{T},\sum_{i=-N_{p}}^{N_{p}}D_{m}\left(i\right)\cdot \cos2\pi i/\hat{T}\right),$$

where $\hat{T}$ was determined from the data in the image, estimated at $\hat{T}=7.1$ pixels.

**Figure 9:**
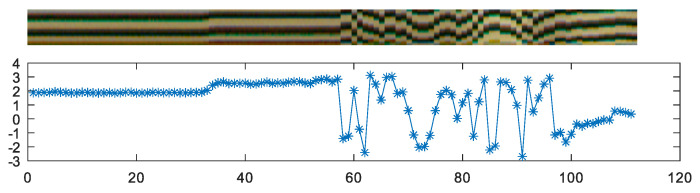
Phase data extracted over time (**top**) with decoded phase value in radian (**bottom**).

Figure 9 illustrates the pattern changing over time (due to the rotation of the object) and the decoded phase value. The period of the extracted signal $\hat{T}$ is defined by a sequence of white and black stripe, which in terms of the target object rotation, equals to a period of 4 for the Gray code (there are two changes per each stripe detected, as shown in Table 2). The main idea is to replace the last two bits of the digitally encoded position $\alpha_{m}$ (4 discrete values) with a continuous value, obtained from $\phi_{m}$. As a result, we get the measurement resolution defined by the phase data signal and avoid ambiguous angle position with the help of Gray code data.

To successfully fuse the data of both sources, we need to align the results—phase data $\phi_{m}$ must be shifted slightly by $\phi_{offset}$ to assure that $\phi_{m}-\phi_{offset}$ equals 0 at the rotation angle, where the third bit of the $\alpha_{m}$ changes value. To emulate the encoder, we then rescale the range of $\phi_{m}$ from $\left[0,2\pi \right)$ to $\left[0,4\right)$ and combine it with the $\alpha_{m}$ that has been stripped of lower two bits (bits set to 0). Considering the $\phi_{m}$ and $\alpha_{m}$ are both affected by the signal noise, we can expect the discrepancy of the two due to the modular nature of the angles. We address that by comparing the resulting combined angle to the position $\alpha_{m}$—since the difference cannot be more than ±2, we can add or subtract 4 to the result to meet the condition.

The described data fusion is performed by executing these steps:

1. $\phi_{m}$ is adjusted with the offset of the Gray code start phase angle and rescaled to have the period of 4: $\phi_{m}^{\prime }=\frac{4}{2\pi }\left[\phi_{m}-\phi_{offset}\right]$,
2. we strip 2 bits from $\alpha_{m}$ (binary AND operation with mask b11100), $\alpha_{m}^{\prime }=\alpha_{m}\;\mathrm{AND}\;b11100$,
3. difference $\phi_{m}^{\prime }+\alpha_{m}^{\prime }+4j-\alpha_{m}$ is wrapped to interval $\left[-2,2\right]$ by adjusting the value of j∈Z,
4. final angular position $\theta_{m}\in \left[0,32\right)\cap R$ is produced: $\theta_{m}=\phi_{m}^{\prime }+\alpha_{m}^{\prime }+4j$.

### 2.6. Final Resolution of the Measurement Results

Since the resolution of the phase data is not explicitly limited, we can estimate it from the noise level in the data. The standard deviation obtained from the experimental measurements for the phase data is limited to $\sigma_{\phi }<0.1$ (radian), which results in a final angular resolution of $0.7^{\circ }$.

**Figure 10:**
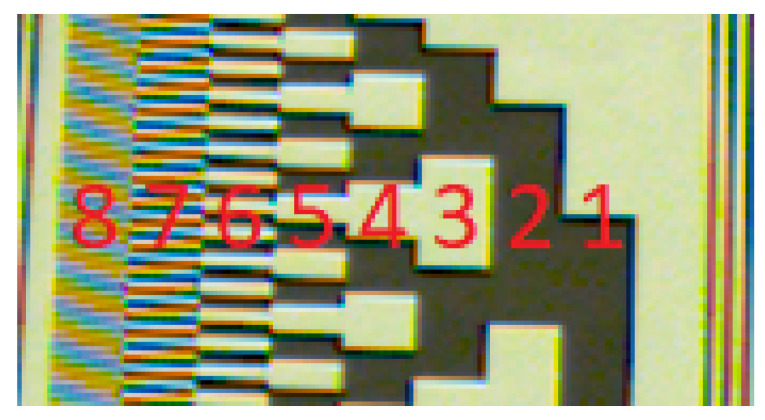
Resolution test sheet.

The comparable angular resolution would be obtained by a 9-bit digital encoder, which would require 128 black and white bars in the finest bit space of the pattern. The standard application of Gray code decoder using the same camera setup would allow only for 7-bit code (resolution of $2.8^{\circ }$), as shown in the resolution test sheet in Figure 10. It can be seen that although 7th bit data still can be regarded as the pattern, the code area with 8th bit data is practically unreadable. Moreover, it is expected that only 6 bits ($5.6^{\circ }$ resolution) would be decodable during dynamic object tracking due to motion blur. The proposed solution therefore provides 4- to 8-fold improvement in angular resolution. This result not only matches but also greatly exceeds the initial requirements.

The linear resolution of the proposed system is also linked to the resolution of the camera—depending on the location of the object in the camera view, it was estimated to the interval between 0.6 and 0.9 mm for the presented application.

### 2.7. System Calibration

In any visual sensing applications, the camera and system calibration is an important step that can not be omitted. In the presented system, it is assumed that the camera is statically mounted in regards to the plane with tracked objects. Therefore, our system requires only two major calibration steps—manual location of three points along paths of tracked objects and determination of marker offsets.

Unlike traditional camera-based object tracking, our approach does not require estimation of extrinsic parameters of the camera. Instead, the effects of lens distortion and projection transformation are integrated into the presented data extraction algorithm. During calibration, the operator is instructed to select 3 well-spaced points along paths of tracked objects. This can either be achieved by moving the tracked object and recording its position or by selecting points along the path directly (if visible to the camera).

Second step deals with determining how the marker pattern was attached to the tracked object. There are three parameters that need to be defined: two linear offsets (marker start offset and maker spacing offset $O_{m}$) and angular offset. These parameters are measured in the actual implementation of the system.

## 3. Application

Over the past few years, the team of Laboratory of Control Systems and Cybernetics organizes competitions (e.g., robot soccer, Lego Mindstorms, drones, SCADA and other automation related tasks) for high school, bachelor and graduate students, where students are given a task that they need to execute better and faster than the other teams (homepage at https://lego-masters.si/). The goal of the tasks is usually more focused on automation and control aspects and less on the mechanics itself, although the best designs are a combination of very good solutions in both of the areas.

Recently, we have decided that a new competition will be organized, presenting a new and attractive task for the competing teams. It is bringing together ideas of the student competitions over the past years and the laboratory’s engagement in FIRA championships years ago [27]. The new sport features a table football and a mixed set of players—creating a cybernetic match with both human and computer players. In order to allow for a competitive play with play strategies extending simple block and kick steps, we think that knowledge of the full system state (ball position, position and angles of the players) is necessary. Multiple teams have already worked on an automated table football game platforms in the past, even resulting in a commercial product [28,29], while other solutions are mostly Master thesis or research platforms [30]. In most cases, the authors focused on realtime ball tracking and omitted the player rods [31], while others did also include partial [32,33] or full player position tracking, as in Reference [34]. While solution presented in [32] relies solely on camera image, authors used no additional markers on the players and were thus limited to measuring only the linear position of them. The solution of the EPFL’s team [34] enables measurement of both the rotation and linear position, but relies on pairs of expensive laser distance sensors in addition to the camera. Our solution relies on using a pair of cameras to track both the ball and the player positions—the system can thus be realized in a compact and unobtrusive fashion.

**Figure 11:**
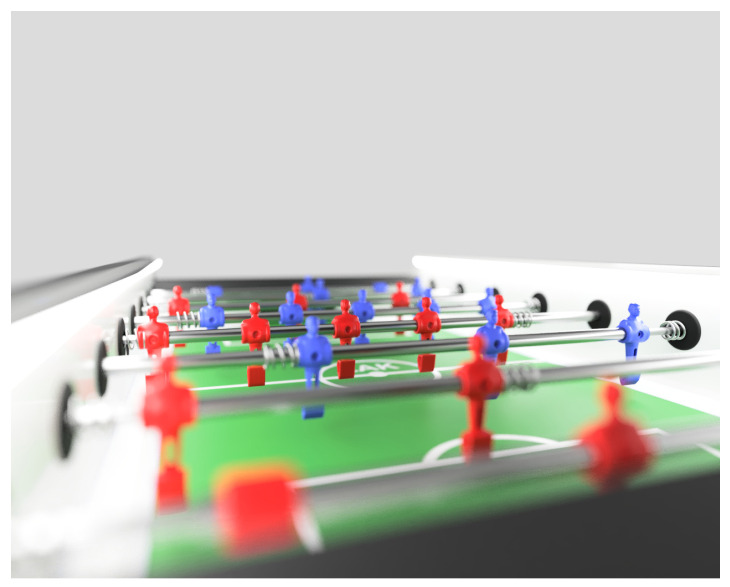
Table football.

The automated table football system requires fully-functional actuator, sensor, and processing sub-systems. The task appears to be simple at first but turns out a real challenge, because it requires robust and accurate tracking of a colored ball and 8 playing rods with players in the field (illustrated in Figure 11) and move the computer-controlled playing rods according to the game rules and strategy. Since we plan to leverage the capabilities of humans and computers on both the perception and actuation, all playing rods (played by human and computer players) need to be tracked.

Therefore, the original intent and requirements for the sensor system introduced in this paper were based on the goal of implementing the described automated table football system. What does seem like a tool for the entertainment, quickly gets a more serious note as soon as the system needs to be implemented in an affordable and robust way. The problem calls for innovative approaches, applicable also to other fields and applications. The paper has presented the approach to track the playing rods, cylindrical targets—each being a 2-DOF (degree of freedom) object, that can be translated (within boundaries) and rotated.

**Figure 12:**
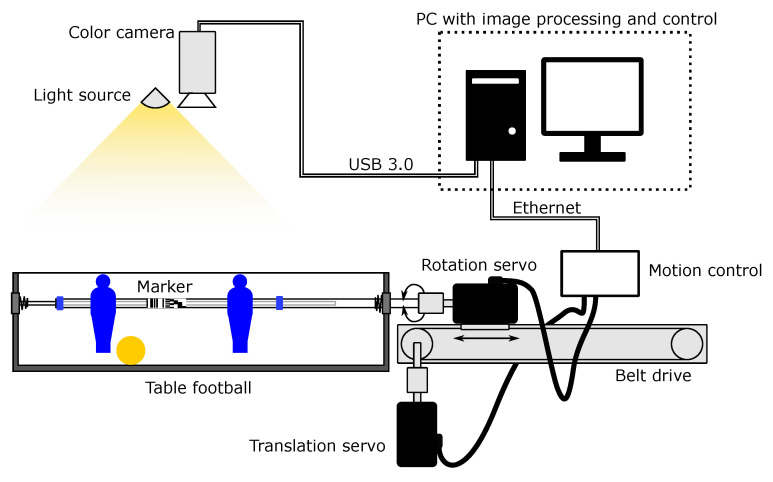
Test setup.

A pair of Basler acA1440-220uc USB 3.0 color video cameras with 1.6 MP resolution (resulting in an image of 1440 by 1080 pixels) and f4mm lenses was positioned over the playing area as shown in Figure 12 (only one of the two cameras is show due to higher intelligibility and transparency of the illustration). A network of video cameras enables us to cover the complete playing area and keep reasonable requirements for the image sensor resolution. Moreover, a multitude of cameras provides additional viewing angles, leveraging the tracking problem in case of mild obstructions. The height and pitch angle of the cameras were determined by manual optimization, where we searched for the low height over the area (to optimize the spatial resolution for object detection and tracking) and improved coverage of the field from multiple angles (e.g., to improve the accuracy of objects recognition due to the overlapping set and the uncertainty of the results in case of partial occlusion of the tracked objects).

The image processing system that is implemented in C++ runs on a desktop PC and uses the Pylon library for capturing images taken by the two cameras. It features the implementation of the presented target tracking approach that is able to track 7 playing rods at a time from a single camera at the frame rate of 200 frames per second. In the next section, we will be presenting the results of only one of the playing rods to improve the readability. Further optimizations of the algorithms are planned in order to extent them to the ball tracking as well.

### 3.1. Experiments

The proposed method was evaluated by conducting experiments in the target application environment. Three tests were executed, the first focusing on the performance of the linear position, the second to the performance of the angular position determination, and the third tests, where both linear and angular positions were tracked. In all cases, the playing rod was actuated in both linear and rotational axis by a closed-loop servo system, shown in Figure 12. A belt-driven linear axis is used as the base platform, moving an additional servo motor, which is coupled to the playing rod via a bi-directional thrust bearing. Both servo motors are controlled via motion control interface PoKeys57CNC, connected to the test PC via Ethernet. The task of the motion control system is to generate control signals for the servo motors in the form of the step and direction pulses. The PoKeys57CNC device is commanded a target position for both motors and built-in motion planner generates motion signals using a trapezoidal velocity curve (constant acceleration and deceleration), which smoothens the motion of the servo motors. The servo motors themselves were separately tuned using the automated self-tuning algorithms. The current commanded position is periodically obtained from the motion control device and compared with the results of the image processing system.

#### 3.1.1. Experiment 1: Tracking Linear Position

**Figure 13:**
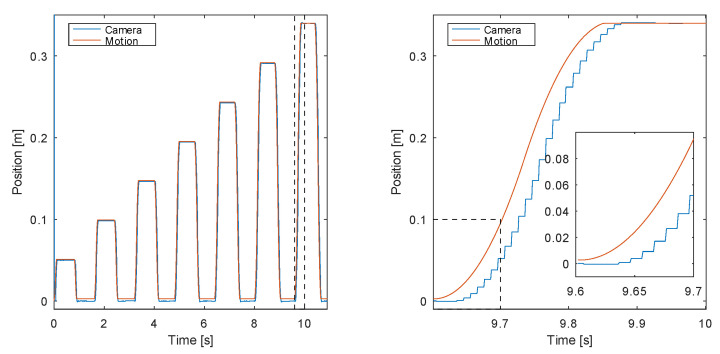
Experiment 1—tracking linear position of the object, 7 step changes were executed (**left**), with a close-up provided on the (**right**).

The aim of the first experiment is to validate the system for tracking the linear position of the target. This step tests the tracking of the marker pattern using the correlation technique described in Section 2.3. Steps of the increasing sizes were programmed for the servo motor on the linear axis, as shown in Figure 13 (left).

The results of the experiment show very good tracking of the linear position of the playing rod. Furthermore, we are observing a response of a non-minimum phase type in the tracked position, which is the side effect of the servo closed-loop system. This effect is shown in the enlarged part of the right graph, where the position, obtained by the proposed system, changes immediately with the commanded motion, but in the opposite direction. After that initial anomaly, the servo motor tracks the commanded position with a relatively constant delay of approx. 25 ms. Since the change (albeit in the opposite direction) immediately follows the commanded position, we can estimate the visual system delay to 1 sample or less (less than 10 ms). Furthermore, the standard deviation of the position error excluding the data with the actual motion was estimated at 0.5 mm, which correlates with the expected resolution of the system.

#### 3.1.2. Experiment 2: Tracking Angular Position

**Figure 14:**
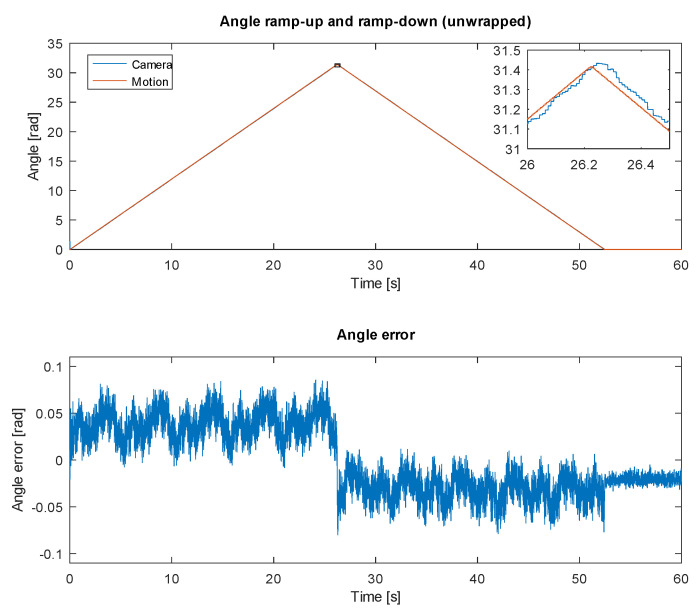
Experiment 2—tracking angular position of the object by observing it rotating 5 revolutions in one direction and 5 revolutions back to initial position (**top**) with resulting angular error (**bottom**).

The second experiment targeted tracking of the angular position via the proposed method. The rotation servo was commanded 5 rotations (angle of 10π) in one direction and back at an angular velocity of 1.2 rad/s. The results are shown in Figure 14. For the illustration purposes, the displayed angle was unwrapped (steps of ±2π due to the results being wrapped to interval $\left[0,2\pi \right\}$ were ignored).

We can observe the angular error to be bound to $\left\{-0.1,0.1\right\}$ interval with a distinctive direction-related offset, which can be contributed to the motion system delay of 25 ms (resulting in the expected offset in the angular error of 1.2rad/s·0.025s=0.03rad). By adjusting for this offset, we can assume the angular error of the tested system to be bound to interval $\left\{-0.03,0.03\right\}$ (less than $2^{\circ }$). The standard deviation of the error was estimated at 0.013 rad in each direction, which corresponds to less than $1^{\circ }$.

Periodic nature (and sawtooth shape) of the angular error indicates a possible improvement by adjusting the position of the marker on the tracked target (there might be a slight discrepancy between the actual marker size and the target circumference).

#### 3.1.3. Experiment 3: Tracking Both Linear and Angular Position

**Figure 15:**
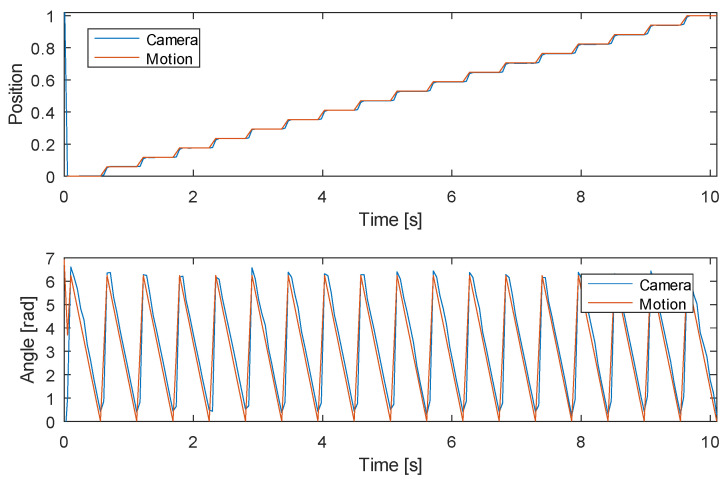
Experiment 3—tracking both linear (**top**) and angular position (**bottom**).

The third experiment’s objective was the validation of all algorithms of the proposed system. While the linear axis was commanded to move in 17 steps between two extreme positions, the rotation axis was commanded to the angle 2π and back to 0 at each step of the linear axis (as shown in Figure 15).

The results show the correct tracking of motion in both axes over the complete range. Moreover, there were no issues detected while tracking fast rotation motion with over 60 rad/s (approx. 600 revolutions per minute) under normal lighting conditions. The system’s capability of tracking even faster motion depends mostly on the illumination system for the cameras—the camera exposure time is namely dictated by the amount of light in the scene. If motion bluring would start presenting itself as a problem, we plan to decrease the exposure time either on the account of noise or adding more lights. However, the system is currently capable of tracking the game played by our fastest student players with no interruptions due to motion blurring. We expect that the slightly lower impulsive velocities of the implemented actuator system in comparison to human players will be compensated with perfect tracking over the complete playground and repeatable and accurate player manipulation.

#### 3.1.4. Tracking Gameplay

**Figure 16:**
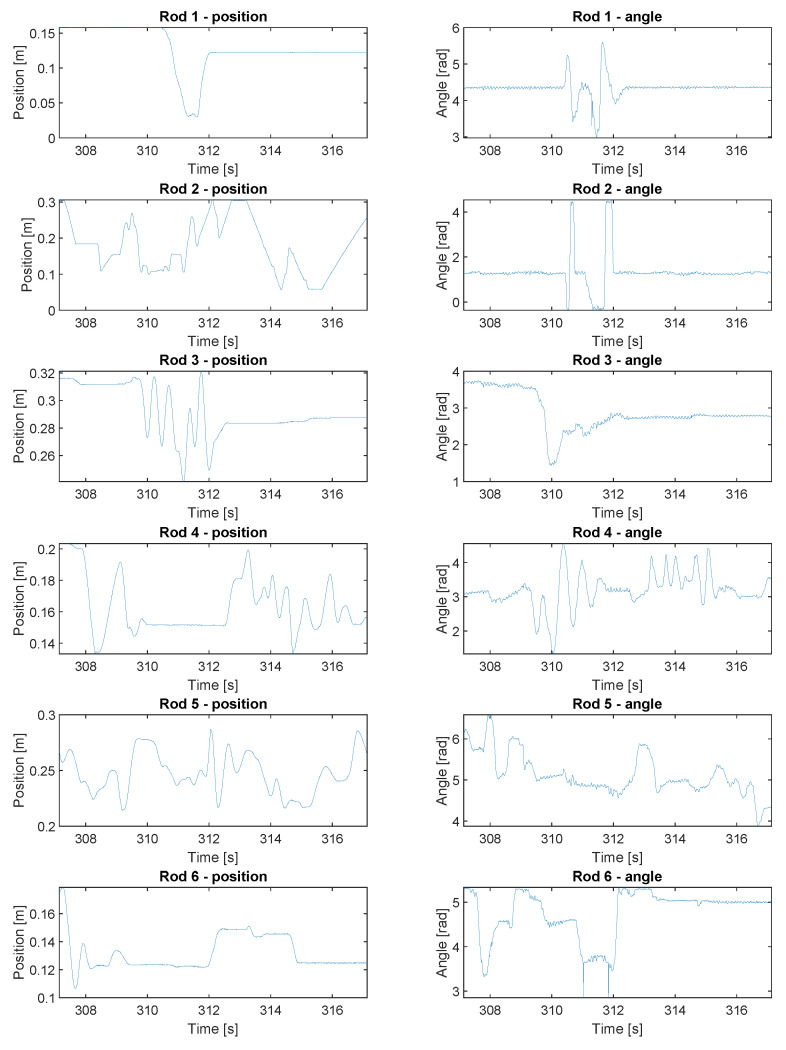
Tracking gameplay—rods positions and angles during a test gameplay.

The system was put into test during a test gameplay between human players and one computer player (on rod 2), which was programmed with a simple block and kick algorithm. The tracking results are shown in Figure 16, where the rods positions and angles are shown. Increased noise level and occasional spikes in the angle results are the result of the system running only with office ceiling luminaries and with frequent obstructions due to human players intervening in the camera’s field of view.

## 4. Conclusions

In this paper, we proposed a novel application of a computer vision system for accurate and fast tracking of the target object’s motion in both the rotation and translation. The non-contact nature allows the sensing element (camera) to be positioned away from the tracked objects, thus covering a wider area for object tracking. This results in not only cleaner implementation in the final application, but also allow multiple objects to be tracked by a single camera, further simplifying the sensory system design. We have provided the results of the experiments, that clearly show the proposed system meeting and even exceeding the design requirements. Further development will focus on improving the computational footprint of the presented system and incorporating the tracking of other objects in the final design, which will allow a single camera system to track all objects needed to support a cyber table football game. The ball tracking is a separate process and is one of the most important ones for a successful automation of the game. In this paper, we focused on determining the rotary-linear transformations of objects (the player positioning system) and omit ball tracking due to the complexity of the latter. Similarly as in player positions, the computer will have the advantage of having real-time overview of the complete state of the system. We expect that computer system with 5–10 ms sampling time will be superior to human (with reaction times in hundredths of milliseconds) in terms of tracking and actuation, but will fail short to unpredictability of human players. A camera-based sensing system, integrated into a unobtrusive overhead pillar, paired with a compact actuator system, and a competitively behaved computer-based player will result in a cost-effective and thus commercially-attractive application of the proposed idea.

## Figures and Tables

**Table 1:** Comparison of common encoder types in terms of mounting types (as shown in Figure 1) and translation compatibility.

Encoder Type	Mounting Type	Highlights	Translation Compatibility
Standard (COTS) encoder	a	Standard industrial components, readily available	Medium, requires mechanical telescopic coupling
Radially mounted incremental encoder	b	Can be mounted along the shaft	Medium, requires shaft features in axial direction
Radially mounted absolute encoder	b	Can be mounted along the shaft	Not compatible
Face mounted magnetic or optical encoder	d	No physical connection needed with the shaft	Low, only limited translation possible
Disk-feature based encoders	e	Common implementation type	Low, only limited translation possible
Resolvers	a, c	Analog angular sensor, mostly discontinued	Low, only limited translation possible
Camera based solutions	b, d, e	Highly flexible, but complex implementation	High, but requires target tracking

**Table 2:** Gray code decoder table for 5-bit code.

$C_{m}$	$\alpha_{m}$	$C_{m}$	$\alpha_{m}$	$C_{m}$	$\alpha_{m}$	$C_{m}$	$\alpha_{m}$
00000	0 ($0^{\circ }$)	01100	8 ($90^{\circ }$)	11000	16 ($180^{\circ }$)	10100	24 ($270^{\circ }$)
00001	1 ($11.25^{\circ }$)	01101	9 ($101.25^{\circ }$)	11001	17 ($191.25^{\circ }$)	10101	25 ($281.25^{\circ }$)
00011	2 ($22.5^{\circ }$)	01111	10 ($112.5^{\circ }$)	11011	18 ($202.5^{\circ }$)	10111	26 ($292.5^{\circ }$)
00010	3 ($33.75^{\circ }$)	01110	11 ($123.75^{\circ }$)	11010	19 ($213.75^{\circ }$)	10110	27 ($303.75^{\circ }$)
00110	4 ($45^{\circ }$)	01010	12 ($135^{\circ }$)	11110	20 ($225^{\circ }$)	10010	28 ($315^{\circ }$)
00111	5 ($56.25^{\circ }$)	01011	13 ($146.25^{\circ }$)	11111	21 ($236.25^{\circ }$)	10011	29 ($326.25^{\circ }$)
00101	6 ($67.5^{\circ }$)	01001	14 ($157.5^{\circ }$)	11101	22 ($247.5^{\circ }$)	10001	30 ($337.5^{\circ }$)
00100	7 ($78.75^{\circ }$)	01000	15 ($168.75^{\circ }$)	11100	23 ($258.75^{\circ }$)	10000	31 ($348.75^{\circ }$)

## References

[1] Dimmler M., Dayer C. (1996). Optical encoders for small drives. IEEE/ASME Trans. Mechatron..

[2] Yang B., Liu Q., Zhang T., Cao Y., Feng Z., Meng G. (2012). Non-contact translation-rotation sensor using combined effects of magnetostriction and piezoelectricity. Sensors.

[3] Lewotsky K. (2019). How to Select the Right Encoder for Your Motion Axis. Motion Control Online.

[4] Kolano K. (2020). Determining the Position of the Brushless DC Motor Rotor. Energies.

[5] Attaianese C., Tomasso G. (2007). Position measurement in industrial drives by means of low-cost resolver-to-digital converter. IEEE Trans. Instrum. Meas..

[6] Genovesi S., Costa F., Borgese M., Dicandia F., Manara G. (2018). Chipless Radio Frequency Identification (RFID) Sensor for Angular Rotation Monitoring. Technologies.

[7] Schwartz M., Etzion T. (1999). The structure of single-track Gray codes. IEEE Trans. Inf. Theory.

[8] Denic D.B., Miljkovic G.S. (2009). Code reading synchronization method for pseudorandom position encoders. Sens. Actuators A Phys..

[9] Das S., Sarkar T.S., Chakraborty B., Dutta H.S. (2016). A simple approach to design a binary coded absolute shaft encoder. IEEE Sens. J..

[10] Dziwinski T. (2015). A novel approach of an absolute encoder coding pattern. IEEE Sens. J..

[11] Sugiyama Y., Matsui Y., Toyoda H., Mukozaka N., Ihori A., Abe T., Takabe M., Mizuno S. (2008). A 3.2 kHz, 14-bit optical absolute rotary encoder with a CMOS profile sensor. IEEE Sens. J..

[12] Palacin J., Valgañon I., Pernia R. (2006). The optical mouse for indoor mobile robot odometry measurement. Sens. Actuators A Phys..

[13] Tresanchez M., Pallejà T., Teixidó M., Palacín J. (2010). Using the image acquisition capabilities of the optical mouse sensor to build an absolute rotary encoder. Sens. Actuators A Phys..

[14] Reinoso O., Payá L. (2020). Special Issue on Visual Sensors. Sensors.

[15] Tomažič S., Dovžan D., Škrjanc I. (2019). Confidence-interval-fuzzy-model-based indoor localization. IEEE Trans. Ind. Electron..

[16] Kim H., Yamakawa Y., Senoo T., Ishikawa M. (2016). Visual encoder: robust and precise measurement method of rotation angle via high-speed RGB vision. Opt. Express.

[17] Bajić J.S., Stupar D.Z., Dakić B.M., Živanov M.B., Nagy L.F. (2014). An absolute rotary position sensor based on cylindrical coordinate color space transformation. Sens. Actuators A Phys..

[18] Wang H., Wang J., Chen B., Xiao P., Chen X., Cai N., Ling B.W.K. (2015). Absolute optical imaging position encoder. Meas. J. Int. Meas. Confed..

[19] Kanno Y., Sato Y. Linear and Angular Position Sensing for Two- Degrees-of-Freedom Motor. Proceedings of the 8th International Conference on Sensing Technology.

[20] Acher O., Nguyen T.L., Lehmann P., Osten W., Gonçalves A.A. (2019). Turning a machine vision camera into a high precision position and angle encoder: NanoGPS-OxyO. Optical Measurement Systems for Industrial Inspection XI.

[21] Doulamis A. (2010). Dynamic tracking re-adjustment: A method for automatic tracking recovery in complex visual environments. Multimed. Tools Appl..

[22] Faessler M., Fontana F., Forster C., Scaramuzza D. Automatic re-initialization and failure recovery for aggressive flight with a monocular vision-based quadrotor. Proceedings of the 2015 IEEE International Conference on Robotics and Automation (ICRA).

[23] Li B., Ouyang W., Sheng L., Zeng X., Wang X. GS3D: An efficient 3D object detection framework for autonomous driving. Proceedings of the 2019 IEEE/CVF Conference on Computer Vision and Pattern Recognition (CVPR).

[24] Panin G., Knoll A. (2006). Fully automatic real-time 3D object tracking using active contour and appearance models. J. Multimed..

[25] Engelberg S., Benjamin H. (2005). Pseudorandom sequences and the measurement of the frequency response. IEEE Instrum. Meas. Mag..

[26] Boyes G. (1980). Synchro and Resolver Conversion.

[27] Matko A.D., Belič A., Klančar G., Blažič S. Robot soccer as a teaching and examining tool. Proceedings of the IEEE Global Engineering Education Conference, EDUCON.

[28] Kleinmotoren geben Tischfußballroboter Schusskraft—Faulhaber. https://www.faulhaber.com/de/maerkte/consumer/tischfussball.

[29] Kneipensportler Starkick Tischfussball-Roboter im Hamburger Kieztest—YouTube. https://www.youtube.com/watch?v=lwxJakdf4zA.

[30] Guenat E., Picard C., Serigado R., Ulrich B. (2012). Automatic Foosball—Concurrent Engineering Project.

[31] Janssen R., De Best J., Van De Molengraft R. (2010). Real-time ball tracking in a semi-automated foosball table. Lecture Notes in Computer Science (Including Subseries Lecture Notes in Artificial Intelligence and Lecture Notes in Bioinformatics).

[32] Myrup A.C., Ørding-Thomsen M. (2007). Software til Automatiseret Bordfodbold—Polyteknisk Midtvejsprojekt.

[33] Automated Foosball Table; Danmarks Tekniske Universitet, Ørsted DTU, Institut for Automation: Kongens Lyngby, Denmark. http://foospmp.myl.dk/.

[34] Riden P. (2016). It’s Only a Game: Robots Defeat Humans on Foosball Playing Field—New Atlas—Robotics. https://newatlas.com/epfl-robot-table-soccer-foosball/44863/.

